# Corpus Cavernosum Abscess Secondary to Traumatic Perforation of Urethral Diverticulum

**DOI:** 10.7759/cureus.7032

**Published:** 2020-02-18

**Authors:** Thomas C Gore, Anna Schepcoff, Domenick Sorresso

**Affiliations:** 1 Internal Medicine, Regional Medical Center Bayonet Point, Hudson, USA

**Keywords:** urethral diverticulum, penile abscess, corpus cavernosum, peptoniphilus, foley catheter, wound infection, wound vac, corporal cavernotomy, urethral perforation

## Abstract

Abscess of the corpus cavernosum is a condition that occurs most commonly as a result of penile injection, priapism, sexually transmitted infections, and trauma. The diagnosis of corpus cavernosum abscess is made through imaging, typically computed tomography (CT) or ultrasound. The preferred method of treatment for corpus cavernosum abscess is incision, drainage, and antibiotic therapy. Urethral diverticulum (UD) is defined as a saccular outpouching of the urethral lumen. We present a unique case of corpus cavernosum abscess secondary to perforation of a UD requiring extensive surgical intervention and resulting in long-term complications.

## Introduction

A corpus cavernosum abscess is a rare condition. Usual etiologies include penile injection of erectile dysfunction medications or illicit drugs, priapism, sexually transmitted infections, and trauma; however, there have been reported cases of idiopathic etiology. The most common causative organisms include Staphylococcus aureus, Streptococci, and Bacteroides [1]. Diagnosis is typically made clinically and through imaging (computed tomography [CT] and ultrasound). The first-line treatment is the use of intravenous (IV) antibiotics and surgical drainage. More conservative treatment, however, includes aspiration in conjunction with IV antibiotics [2-3].

Urethral diverticulum (UD) is the formation of a saccular outpouching of the urethral lumen. Symptoms may include recurrent urinary tract infections, incontinence, hematuria, and dysuria [4]. We present a unique case of a corpus cavernosum abscess secondary to perforation of a UD through self-catheterization.

## Case presentation

**Figure 1:**
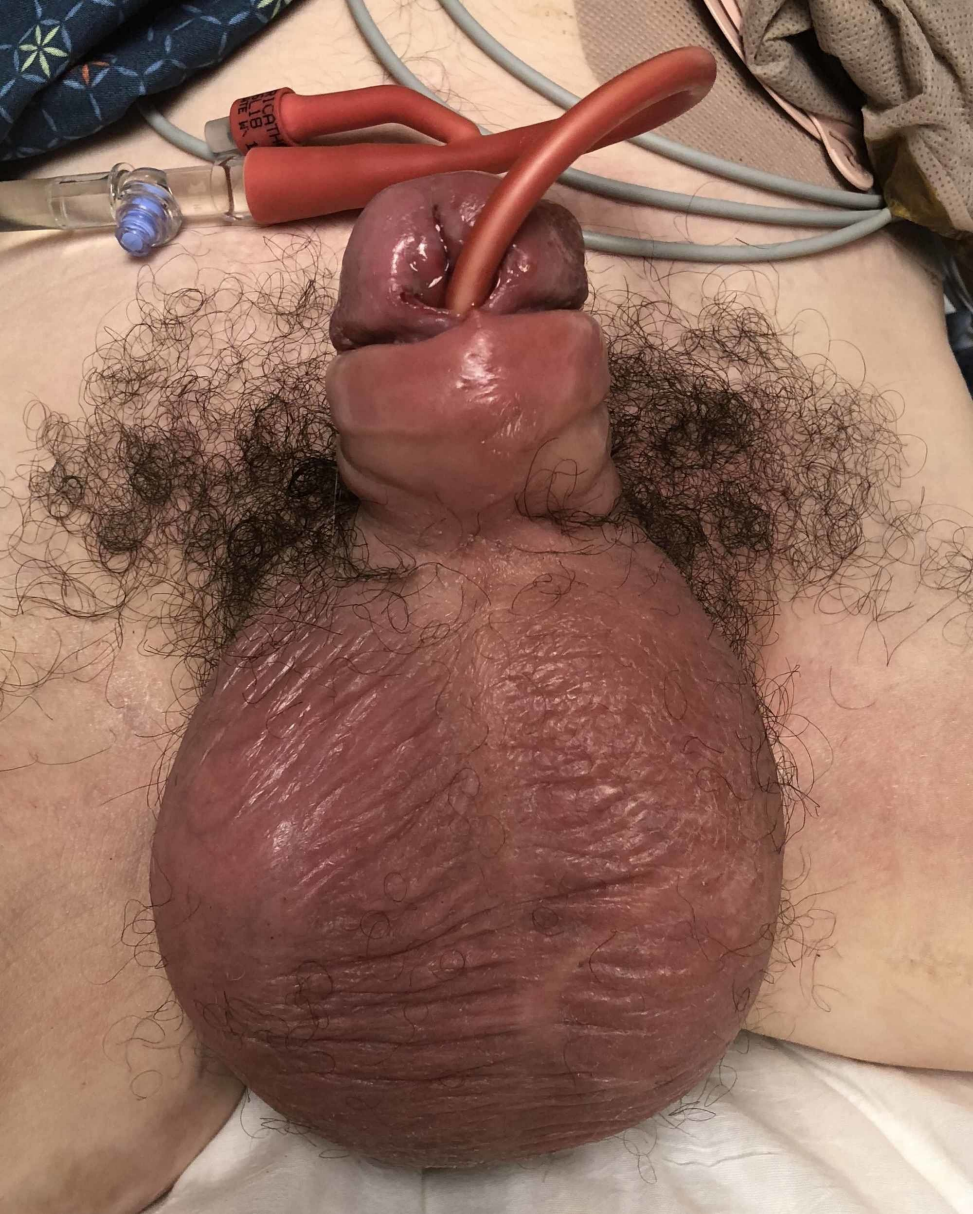
Patient at presentation exhibiting penile and scrotal erythema, priapism, and paraphimosis.

Our patient is a 34-year-old Caucasian male who developed an abscess in the corpus cavernosum secondary to perforation of a UD. Due to a motor vehicle accident, which happened 20 years ago, the patient is a paraplegic with a neurogenic bladder for which he self-catheterizes. The patient reported that during self-catheterization the day prior to presentation, he met resistance and heard a “popping” sound. At presentation, physical examination revealed a diffusely erythematous penis and scrotum with priapism and paraphimosis (Figure 1).

**Figure 2:**
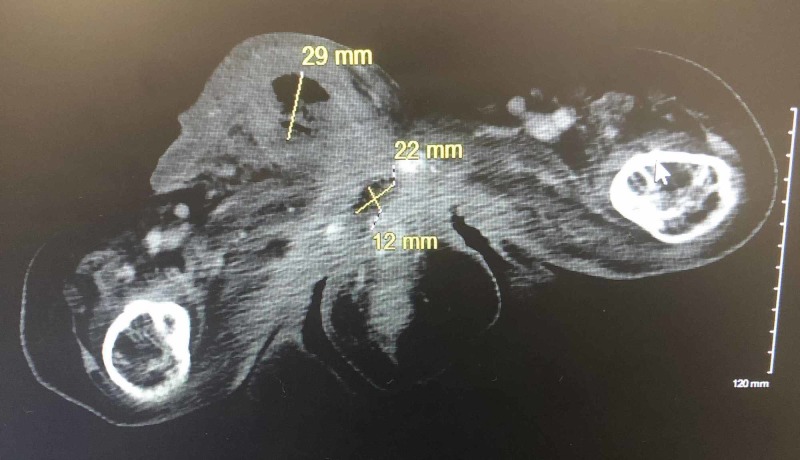
Demonstration of corpus cavernosum abscess with secondary fluid collection on computed tomography (CT).

A CT scan revealed a 7.8 x 2.9 cm abscess within the penile shaft along with a second fluid collection measuring 2.2 x 1.2 cm inferior to the pubic symphysis, causing bladder outlet obstruction (Figure 2).

In the emergency room, a bedside aspiration yielded a small amount of blood noted to be too viscous for arterial blood gas measurement. An emergent groin and scrotal exploration with corporal cavernosum dissection was performed by a urologist, with identification of perforated UD. Postoperatively, the patient was transferred to the intensive care unit and started on IV fluids, vancomycin, and cefepime. Clindamycin was later added per infectious disease consultation recommendations.

**Figure 3:**
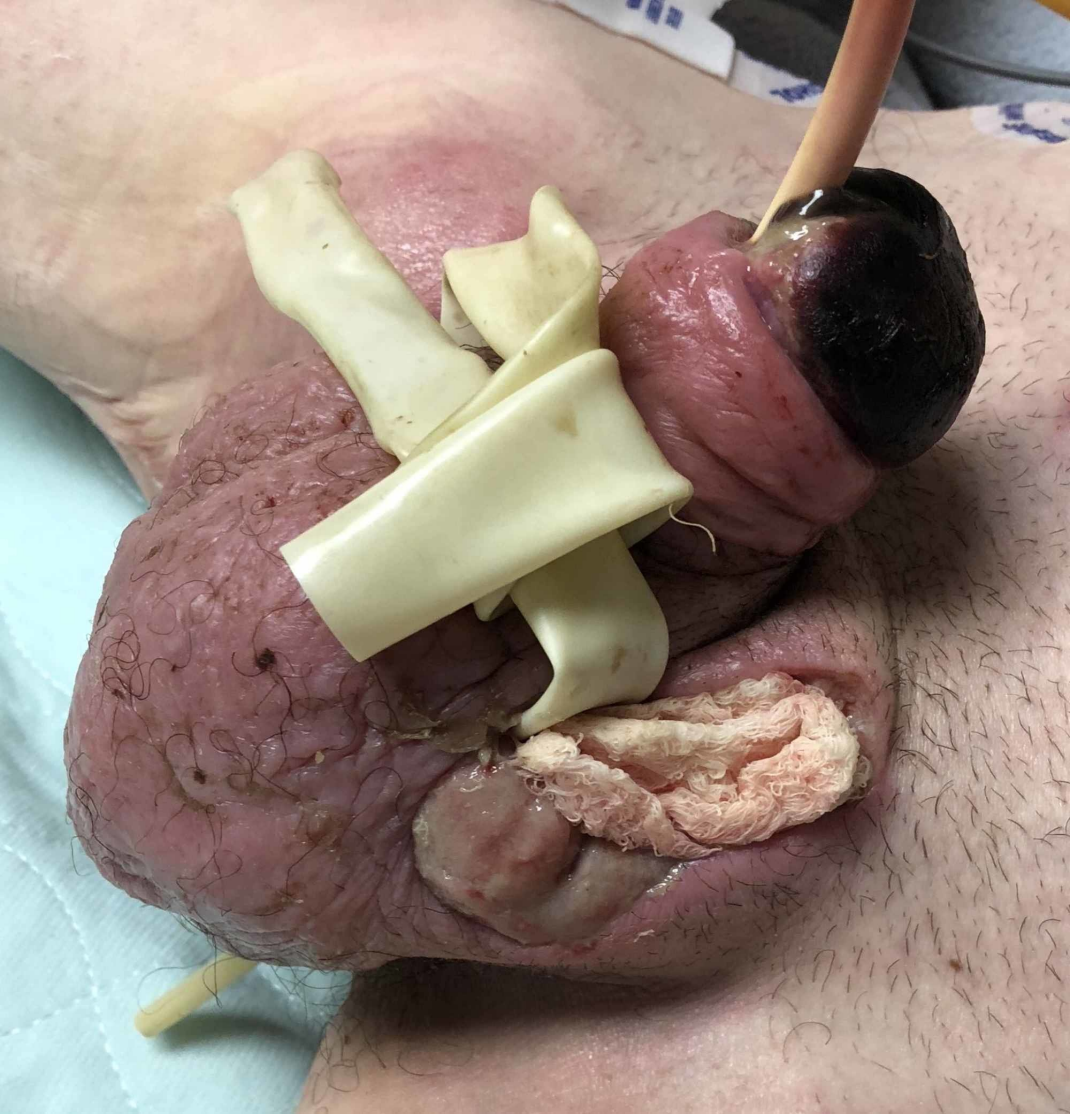
Patient after undergoing repeated exploration with washout, right testicular thigh pouch creation with relocation of the right testicle into the pouch, and bilateral corporal cavernostomies.

Two days after admission, due to crepitus and the development of necrotic tissue, the patient underwent (i) repeated exploration with washout, (ii) right testicular thigh pouch creation with relocation of the right testicle into the pouch, and (iii) bilateral corporal cavernostomies with continuation of current antibiotics (Figure 3).

Four days after admission, the patient’s blood and surgical cultures grew Peptoniphilus asaccharolyticus and Corynebacterium species, respectively. Antibiotics were subsequently adjusted to ceftriaxone and metronidazole. 

Over the course of the next four days, the patient underwent multiple debridement procedures due to the continued development of necrotic tissue. A suprapubic catheter was inserted, whereas a Foley catheter remained in place due to discontinuity between the penile and bulbar urethra. A vacuum-assisted closure (VAC) was performed to promote wound healing. Due to continued necrotic tissue development, partial penectomy with perineal urethrostomy was considered by the urologist. However, the surgery was not performed out of concern for hypoperfusion and poor postoperative healing. Our patient was discharged home with the Foley catheter, suprapubic catheter, and wound VAC in place. He was prescribed sulfamethoxazole/trimethoprim at discharge.

## Discussion

While cases of corpus cavernosum abscesses are uncommon, our case is particularly rare due to the fact that it occurred from traumatic perforation of a UD. There have been documented cases of abscesses secondary to penile injection, priapism, sexually transmitted infections, and even extension of perianal or abdominal abscesses [1]. While there have been documented cases of abscesses secondary to various types of trauma, we did not identify any case that occurred due to perforation of a UD. Another aspect that makes our case unique is that Peptoniphilus asaccharolyticus, a gram-positive anaerobic bacteria communal to the vagina and gut, was found in the blood cultures. Polymicrobial bloodstream infections with Peptoniphilus species have been reported in cases of complex abscesses of the abdomen and pelvis. Meanwhile, bloodstream infections with Peptoniphilus species alone were cited to be more common in immunocompromised patients with soft tissue infections [5].

This case was further complicated by paraphimosis restricting blood supply to the glans penis and bladder outlet obstruction through secondary fluid collection inferior to the pubic symphysis. Due to these complications, the patient underwent extensive surgical intervention as compared with the preferred treatment of simple incision and drainage. Our patient required complete groin and scrotal exploration with corporal cavernosum dissection. Similar cases of periurethral abscess in the presence of urethral defect with subsequent development of necrotic tissue have been reported and also required extensive surgical exploration, wide debridement, and, in some cases, corporal cavernostomy [6-7]. As stated earlier, the more conservative treatment method of aspiration in conjunction with IV antibiotics is sometimes used to reduce the risk of long-term postoperative complications such as erectile dysfunction [1,3]. This treatment method was not an option in our particular case due to the emergent nature of presentation.

Ultimately, our patient did recover from his corpus cavernosum abscess after multiple surgeries and debridements secondary to continued formation of necrotic tissue. The glans penis and distal third of the penile shaft were lost secondary to necrosis, resulting in the patient having permanent erectile dysfunction and a chronic suprapubic catheter.

## Conclusions

Corpus cavernosum abscess can occur secondary to perforation of a UD. While typically treated with simple incision and drainage in conjunction with IV antibiotic therapy, corpus caverosum abscess may require more extensive surgical intervention in the setting of complications such as urethral defect and bladder outlet obstruction. Patients who require more extensive surgical intervention, particularly dissection of the corporal cavernosum, are more likely to suffer long-term complications such as development of scar tissue and erectile dysfunction.
